# *Streptococcus pneumoniae* nasal carriage patterns with and without common respiratory virus detections in households in Seattle, WA, USA before and during the COVID-19 pandemic

**DOI:** 10.3389/fped.2023.1198278

**Published:** 2023-07-07

**Authors:** Julia C. Bennett, Anne Emanuels, Jessica Heimonen, Jessica O'Hanlon, James P. Hughes, Peter D. Han, Eric J. Chow, Constance E. Ogokeh, Melissa A. Rolfes, Christine M. Lockwood, Brian Pfau, Timothy M. Uyeki, Jay Shendure, Samara Hoag, Kairsten Fay, Jover Lee, Thomas R. Sibley, Julia H. Rogers, Lea M. Starita, Janet A. Englund, Helen Y. Chu

**Affiliations:** ^1^ Department of Medicine, University of Washington, Seattle, WA, United States; ^2^ Department of Epidemiology, University of Washington, Seattle, WA, United States; ^3^ Department of Biostatistics, University of Washington, Seattle, WA, United States; ^4^ Brotman Baty Institute for Precision Medicine, University of Washington, Seattle, WA, United States; ^5^ Military and Health Research Foundation, Laurel, MD, United States; ^6^ Communicable Disease Epidemiology and Immunizations Section, Prevention Division, Public Health – Seattle & King County, Seattle, WA, United States; ^7^ Centers for Disease Control and Prevention, Atlanta, GA, United States; ^8^ Department of Laboratory Medicine and Pathology, University of Washington, Seattle, WA, United States; ^9^ Department of Genome Sciences, University of Washington, Seattle, WA, United States; ^10^ Student Health Services, Seattle Public Schools, Seattle, WA, United States; ^11^ Vaccine and Infectious Disease Division, Fred Hutchinson Cancer Center, Seattle, WA, United States; ^12^ Seattle Children’s Research Institute, Department of Pediatrics, University of Washington, Seattle, WA, United States

**Keywords:** streptococcus pneumoniae, pneumococcal, nasal carriage, respiratory, viruses, COVID-19 pandemic

## Abstract

**Background:**

Respiratory viruses might influence *Streptococcus pneumoniae* nasal carriage and subsequent disease risk. We estimated the association between common respiratory viruses and semiquantitative *S. pneumoniae* nasal carriage density in a household setting before and during the COVID-19 pandemic.

**Methods:**

From November 2019–June 2021, we enrolled participants in a remote household surveillance study of respiratory pathogens. Participants submitted weekly reports of acute respiratory illness (ARI) symptoms. Mid-turbinate or anterior nasal swabs were self-collected at enrollment, when ARI occurred, and, in the second year of the study only, from household contacts after SARS-CoV-2 was detected in a household member. Specimens were tested using multiplex reverse-transcription PCR for respiratory pathogens, including *S. pneumoniae*, rhinovirus, adenovirus, common human coronavirus, influenza A/B virus, respiratory syncytial virus (RSV) A/B, human metapneumovirus, enterovirus, and human parainfluenza virus. We estimated differences in semiquantitative *S. pneumoniae* nasal carriage density, estimated by the inverse of *S. pneumoniae* relative cycle threshold (Crt) values, with and without viral detection for any virus and for specific respiratory viruses using linear generalized estimating equations of *S. pneumoniae* Crt values on virus detection adjusted for age and swab type and accounting for clustering of swabs within households.

**Results:**

We collected 346 swabs from 239 individuals in 151 households that tested positive for *S. pneumoniae* (*n* = 157 with and 189 without ≥1 viruses co-detected). Difficulty breathing, cough, and runny nose were more commonly reported among individuals with specimens with viral co-detection compared to without (15%, 80% and 93% vs. 8%, 57%, and 51%, respectively) and ear pain and headache were less commonly reported (3% and 26% vs. 16% and 41%, respectively). For specific viruses among all ages, semiquantitative *S. pneumoniae* nasal carriage density was greater with viral co-detection for enterovirus, RSV A/B, adenovirus, rhinovirus, and common human coronavirus (*P* < 0.01 for each). When stratified by age, semiquantitative *S. pneumoniae* nasal carriage density was significantly greater with viral co-detection among children aged <5 (*P* = 0.002) and 5–17 years (*P* = 0.005), but not among adults aged 18–64 years (*P* = 0.29).

**Conclusion:**

Detection of common respiratory viruses was associated with greater concurrent *S. pneumoniae* semiquantitative nasal carriage density in a household setting among children, but not adults.

## Introduction

*Streptococcus pneumoniae* remains an important cause of morbidity and mortality among children and adults globally despite the availability of effective pneumococcal conjugate vaccines (PCVs) covering ≥10 *S. pneumoniae* serotypes (1, 2). *S. pneumoniae* nasopharyngeal carriage is a necessary precursor of invasive pneumococcal disease (IPD), although disease risk is influenced by a combination of host, pathogen, and environmental factors and in the majority of cases pneumococcal carriage does not result in disease (3).

Respiratory virus infection is one host factor influencing *S. pneumoniae* carriage and disease risk. Influenza virus infection and risk of increased pneumococcal carriage density and disease has been well-established and investigated in animal (4–6), modeling (7), ecological (8–20), and individual-level epidemiologic (21, 22) studies. Ecologic and epidemiologic studies have also associated infection with respiratory syncytial virus (RSV) and, to a lesser extent, rhinovirus, adenovirus, human metapneumovirus, and human parainfluenza virus with increased pneumococcal disease risk (10–12, 15, 17–20, 23–26).

Increased pneumococcal carriage density has been found to be associated with multiple common respiratory viruses, including rhinovirus, adenovirus, influenza virus, RSV, and common human coronavirus, although viruses and populations investigated varied across studies. Most studies were among children with acute respiratory illness (ARI) or pneumonia (21, 27–30), fewer studies included asymptomatic children (30–32), and one study to our knowledge included adults with ARI (21).

To date, studies describing the association between respiratory viruses and *S. pneumoniae* carriage have been conducted primarily among children and most have evaluated a small number of respiratory viruses co-detected. We aimed to estimate the association between eight common respiratory viruses and semiquantitative *S. pneumoniae* carriage density and to describe symptom profiles with and without viral co-detection among individuals of all ages in a community-based surveillance study among households in Seattle, Washington, USA before and during the COVID-19 pandemic.

## Materials and methods

### Study design, data collection, and laboratory testing

From November 2019–June 2021, a convenience sample of participants consented and enrolled in a prospective, longitudinal household surveillance study of respiratory pathogens in the Seattle metropolitan area as part of the Seattle Flu Study (33). The study design, recruitment, eligibility, and data collection have been previously described (34). Briefly, households with ≥3 individuals and ≥1 child aged 3 months through 17 years were eligible and recruited from elementary and middle schools. Household members of all ages were eligible. One adult, designated as the household reporter, submitted weekly reports online of ARI symptoms for the entire household. ARI was defined as new or worsening acute cough or the presence of two or more other respiratory symptoms (sore throat, muscle or body aches, headache, fatigue, ear pain, sweats, fever, runny nose, chills, difficulty breathing, nausea, rash, and diarrhea).

Mid-turbinate (used during year 1 of the study from November 2019–July 2020) or anterior nasal (used during year 2 of the study from August 2020–June 2021) swabs were self-collected or collected by a parent or guardian at home. Self-swabs were collected at enrollment for all household members and once per participant when each ARI occurred. Additionally, in the second year of the study only (from August 2020–June 2021), when SARS-CoV-2 was detected among an enrolled household member, both symptomatic and asymptomatic household contacts collected self-swabs 4, 6, 8, 10, 12 and 14 days following the positive SARS-CoV-2 case. Among symptomatic individuals, clinical data were collected from individuals or a parent or guardian at swab collection and a one-week follow-up questionnaire collected data on symptoms, illness impact on school and/or work, and health care seeking. Symptom data were not collected for baseline swabs at enrollment or for household contacts of SARS-CoV-2 cases. Data were collected using Research Electronic Data Capture (*REDCap*) (35, 36).

Home self-collected swabs were mailed to the Northwest Genomics Center at the University of Washington at ambient temperature in universal transport media during year 1 of the study and as dry swabs in year 2 of the study. Respiratory pathogens were detected using arrayed reverse-transcription polymerase chain reaction (RT-qPCR) following total nucleic acids extraction (37). The assay has been assessed for accuracy of *S. pneumoniae* detection against control samples, used proprietary primer sets, and the target gene for identifying *S. pneumoniae-*positive specimens was *hflB* (Thermo Fisher Scientific assay ID Ba06439619_s1) (38). The array contained assays specific to enterovirus to avoid rhinovirus cross-reactivity but the rhinovirus assay had cross-reactivity with enterovirus and some rhinovirus detections may reflect enterovirus. Specimens with both rhinovirus and enterovirus detected were considered positive for both viruses in this analysis. Also, in year 2 of the study the rhinovirus assay was expanded to include additional rhinovirus types. *S. pneumoniae* relative cycle threshold (Crt) values, an alternative to cycle threshold values that is used specifically by the OpenArry platform, were based on the amplification curve only rather than all curves for a specific target (39). We used the inverse of *S. pneumoniae* Crt values as an estimation of semiquantitative *S. pneumoniae* nasal carriage density. *S. pneumoniae* serotyping was not performed.

The University of Washington Institutional Review Board approved this study. All participants completed informed consent (or assent and parental or guardian consent for participants <18 years of age at enrollment).

### Data analysis

We described the proportion of all swabs collected with *S. pneumoniae* and/or any virus detected over the study period overall and by age group. All other analyses only included results from swabs with *S. pneumoniae* detected with or without the following respiratory viruses: rhinovirus, adenovirus, common human coronavirus, influenza A/B virus, RSV A/B, human metapneumovirus, enterovirus, and human parainfluenza virus. From January 1, 2020 onward, specimens were additionally tested for SARS-CoV-2. We considered individuals to be symptomatic if they reported any of the following at either sample collection or on the one-week follow-up questionnaire: difficulty breathing, chills, sweats, cough, ear pain, fatigue, fever, headache, muscle and/or body aches, runny nose, or sore throat.

We conducted a cross-sectional analysis of swabs from testing events with *S. pneumoniae* detected with a respiratory virus co-detected (cases) compared to *S. pneumoniae*-positive swabs without a respiratory virus co-detected (controls). We estimated the difference in *S. pneumoniae* Crt values with and without viral co-detection for any virus and for specific respiratory viruses using linear generalized estimating equations (GEE) of *S. pneumoniae* Crt values on virus detection adjusted for continuous age and swab type (mid-turbinate vs. anterior nasal) and accounting for clustering of persons with positive swabs within households (40). Analyses were adjusted for swab type due to the change in swabs used during the study period. For sensitivity analyses we (1) estimated differences in *S. pneumoniae* Crt values with and without viruses detected for symptomatic testing events and additionally adjusted for days since symptom onset, (2) stratified the analysis by pre- and post-implementation of COVID-19 restrictions (beginning in March 2020), and (3) estimated differences in *S. pneumoniae* Crt values with and without viruses detected excluding repeat swabs conducted by participants within 30 days. Secondary analyses included estimating differences in *S. pneumoniae* Crt values with and without any virus by participant age category (children aged <5 years, aged 5–17 years, and adults aged 18–64 years) and differences in *S. pneumoniae* Crt values with and without influenza A/B virus adjusted for current season influenza vaccination status at the time of testing. Analyses were conducted in R (R-4.1.1, R Core Team, 2021).

## Results

From November 2019–June 2021 we collected 4630 swabs from 1,700 unique individuals in 437 households. Of these, 346 (8%) were positive for *S. pneumoniae* and 773 (17%) were positive for any respiratory virus (excluding SARS-CoV-2). Viral detection was greatest during the 2019–early 2020 winter season, declined substantially following COVID-19 pandemic restrictions beginning in March 2020, began to increase during winter 2021, and remained high during the spring and summer months of 2021 through the end of the study in June 2021. Conversely, detection of *S. pneumoniae* was lower and declined to a lesser degree during the COVID-19 pandemic. Among all swabs collected (excluding swabs from household contacts of SARS-CoV-2 cases, which were collected in the second year of the study only), 30% of swabs were positive for any virus (excluding SARS-CoV-2) and 12% were positive for *S. pneumoniae* prior to March 2020 vs. 10% and 6%, respectively, from March–December 2020 and 21% and 5%, respectively, from January–June 2021, although these proportions are not adjusted for the change from mid-turbinate to anterior nasal swabs or the age distribution over time (Supplementary Figure S1). Trends in viral and *S. pneumoniae* detection over the study period were generally similar by age group and the age distribution did not change meaningfully over the study period. One exception was that *S. pneumoniae* detection was greatest among children and prior to the COVID-19 pandemic, the proportion of swabs with any virus only was similar to the proportion with *S. pneumoniae* and any virus for children aged <5 years, whereas for older children and adults a lower proportion of pre-COVID-19 pandemic swabs had *S. pneumoniae* with any virus co-detected relative to any virus only (Supplementary Figure S2). Although we detected SARS-CoV-2 in 35 specimens, none of these had *S. pneumoniae* and SARS-CoV-2 co-detected.

Subsequent analyses were restricted to 346 swabs from 239 unique individuals in 151 households that tested positive for *S. pneumoniae* (*n* = 157 swabs (45%) with and 189 (55%) without one or more viruses detected) (Table 1). The median time between swab collection for individuals with multiple swabs included in the analysis was 42 (range: 1–540 days, interquartile range: 14–103). Approximately half (49%) of participants were male, majority (79%) were White, and most were children (40% children aged <5 years, 31% children aged 5–17 years, and 29% adults aged 18–64 years). Over half (68%) of included households had a child aged <5 years and median household size was 4 individuals (range: 3–7). During the entire study period a median of one swab (range: 1–11) per participant and one swab (1–17) per household were positive for *S. pneumoniae*. Over half (57%) of *S. pneumoniae-*positive swabs without viral co-detection were baseline swabs collected at enrollment, relative to 31% of swabs with viral codetection and 19% of swabs with and 12% of swabs without viral co-detection were collected from household contacts of SARS-CoV-2 positive cases.

**Table 1:** Demographic characteristics of unique participants and households that tested positive for *S. pneumoniae* ≥1 times from November 2019–June 2021.

Total participants, *N*	239
Age at enrollment (years), median (range)	6.1 (0.25–63.2)
Male sex assigned at birth^a^, *N* (%)	116 (48.5%)
Race, *N* (%)	
American Indian/Alaska Native	0 (0%)
Asian	13 (5.4%)
Black	1 (0.4%)
Native Hawaiian or other Pacific Islander	0 (0%)
White	189 (79.1%)
Multiple Races^b^	22 (9.2%)
Unknown	14 (5.9%)
Hispanic ethnicity, *N* (%)	20 (8.4%)
One or more comorbidities^c^, *N* (%)	18 (7.5%)
*Children aged <18 years (N = 171)*	*8 (4.7%)*
*Adults aged 18–64 years (N = 68)*	*10 (14.7%)*
Smokes cigarettes^d^, *N* (%)	1 (0.4%)
*S. pneumoniae*-positive swabs included in analysis, per participant^e^, median (range)	1 (1–11)
Total households, *N*	151
Household with child aged <5 years, *N* (%)	103 (68.2%)
Household size, median (range)	4 (3–7)
*S. pneumoniae*-positive swabs included in analysis per household^e^, median (range)	1 (1–17)
Households with *S. pneumoniae*-positive swabs both pre- and post-March 2020, *N* (%)	29 (19.2%)

^a^ Sex assigned at birth missing for one participant.

^b^ Multiple races includes Asian and White (*n* = 18), White and other (*n* = 2), Native Hawaiian or other Pacific Islander and White (*n* = 1), and Black and White (*n* = 1).

^c^ Comorbidities include asthma, bronchitis, cancer, chronic obstructive pulmonary disease (COPD)/ emphysema, diabetes, and heart disease (history of heart attack or heart failure).

^d^ One participant that reported smoking cigarettes was 38 years of age.

^e^ Number of S. pneumoniae-positive swabs included in analysis per participant and per household are for the entire study period November 2019–June 2021.

Among all *S. pneumoniae-*positive swabs, rhinovirus (*n* = 87; 25%) was the most frequently detected virus, followed by common human coronavirus [*n* = 30 (9%)], adenovirus [*n* = 23 (7%)], influenza A/B virus [*n* = 8 influenza A and *n* = 9 influenza B virus (5%)], RSV A/B [*n* = 9 RSV A and *n* = 5 RSV B (4%)], human metapneumovirus (*n* = 6 (2%)), human parainfluenza [*n* = 5 (1%)], and enterovirus [*n* = 4 (1%)]. Among swabs with *S. pneumoniae* and at least one virus, only 17% had ≥2 viruses co-detected (25 with 2 viruses co-detected and 1 with 3 and 4 viruses co-detected each) and these were mostly (69%) among children aged <5 years. The distribution of viruses co-detected with *S. pneumoniae* varied by age group (Supplementary Figure S3). The proportion of all *S. pneumoniae*-positive swabs with common human coronavirus detected was greater among adults relative to children and no swabs among adults had *S. pneumoniae* co-detected with human parainfluenza.

Among symptomatic individuals, days since symptom onset were similar for specimens with and without viral co-detection (median: 3 days, Table 2). Among individuals reporting any symptoms, difficulty breathing, cough, and runny rose were more commonly reported among specimens with viral co-detection (15%, 80% and 93% vs. 8%, 57%, and 51%, respectively) whereas ear pain and headache were less commonly reported among specimens with compared to without viral co-detection (3% and 26% vs. 16% and 41%, respectively). Fatigue, fever, chills or sweats, muscle and/or body aches, and sore throat were similar between specimens with and without viral co-detection (Table 2 and Supplementary Figure S4). Reported symptoms profiles differed by age group (Supplementary Table S1). Cough, fever and runny nose were more commonly reported among symptomatic children aged <5 (75%, 59%, and 90%, respectively) and 5–17 years (77%, 63%, and 71%) compared to adults aged 18–64 years (60%, 36%, and 68%) while difficulty breathing, headache, and muscle and/or body aches were more commonly reported among adults (24%, 68%, and 40%) compared to children aged <5 (12%, 6%, and 10%, respectively) and 5–17 years (6%, 40%, and 23%). Ear pain and fatigue were reported for a similar proportion of symptomatic adults (12% and 76%) and children aged 5–17 years (11% and 74%), relative to younger children (2% and 47%).

**Table 2:** Characteristics of participants, symptomatic events, and testing results included in analysis.

	Any virus & *S. pneumoniae* (*N* = 157)	*S. pneumoniae* only (*N* = 189)	Total (*N* = 346)
Baseline enrollment swab, *N* (%)	50 (31.2%)	108 (57.1%)	158 (45.7%)
SARS-CoV-2 household contact, *N* (%)	19 (12.1%)	35 (18.5%)	54 (15.6%)
Symptomatic, *N* (%)			
Yes^a^	74 (47.1%)	37 (19.6%)	111 (32.1%)
Days since symptom onset, median (IQR^b^)	3.0 (2.0, 4.0)	3.0 (1.5, 3.0)	3.0 (2.0, 4.0)
Influenza-like illness (ILI)^c^,^d^	33 (44.6%)	16 (43.2%)	49 (44.1%)
Difficulty breathing^c^	11 (14.9%)	3 (8.1%)	14 (12.6%)
Chills or sweats^c^	22 (29.7%)	9 (24.3%)	31 (27.9%)
Cough^c^	59 (79.7%)	21 (56.8%)	80 (72.1%)
Ear pain^c^	2 (2.7%)	6 (16.2%)	8 (7.2%)
Feeling more tired than usual^c^	45 (60.8%)	24 (64.9%)	69 (62.2%)
Fever/feeling feverish^c^	40 (54.1%)	21 (56.8%)	61 (55.0%)
Headache^c^	19 (25.7%)	15 (40.5%)	34 (30.6%)
Muscle and/or body aches^c^	16 (21.6%)	7 (18.9%)	23 (20.7%)
Runny nose^c^	69 (93.2%)	19 (51.4%)	88 (79.3%)
Sore throat^c^	28 (37.8%)	15 (40.5%)	43 (38.7%)
Absent from work or school^c^	32 (43.2%)	18 (48.6%)	50 (45.0%)
Sought medical care^c^,^e^	12 (16.2%)	9 (24.3%)	21 (18.9%)
Unknown^f^	83 (52.9%)	152 (80.4%)	235 (67.9%)
Received current season influenza vaccine at time of testing^g^, *N* (%)			
Yes	116 (73.9%)	134 (70.9%)	250 (72.3%)
No	24 (15.3%)	43 (22.8%)	67 (19.4%)
Unknown	13 (8.3%)	9 (4.8%)	22 (6.4%)
Ineligible^h^	4 (2.5%)	3 (1.6%)	7 (2.0%)
*S. pneumoniae* Crt, median (IQR^b^)	17.3 (14.0, 24.3)	23.5 (18.8, 25.9)	21.1 (15.8, 25.7)
Number of viruses detected, median (range)	1 (1–4)	NA	NA
Swab type^i^, *N* (%)			
Mid-turbinate	118 (75.2%)	124 (65.6%)	242 (69.9%)
Anterior nasal	39 (24.8%)	65 (34.4%)	104 (30.1%)

^a^ Individuals considered to be symptomatic if they reported any of the following at either sample collection or on the one-week follow-up questionnaire: difficulty breathing, chills, sweats, cough, ear pain, fatigue, fever, headache, muscle and/or body aches, runny nose, or sore throat.

^b^ Interquartile range.

^c^ Proportions for specific symptoms, absence from work or school, and sought medical care are among those reporting any symptoms.

^d^ ILI includes fever with cough and/or sore throat.

^e^ Sought medical care unknown for one testing event.

^f^ Swabs with unknown symptom status include baseline enrollment swabs and swabs from household contacts after SARS-CoV-2 was detected in a household member (in the second year of the study only).

^g^ At the time of testing, had received 2019–2020 influenza vaccine for swabs collected between November 2019–July 2020 and 2020–2021 influenza vaccine for swabs collected between August 2020–June 2021.

^h^ Aged <6 months at the time of testing.

^i^ Mid-turbinate swabs used from November 19, 2019–July 22, 2020 and anterior nasal swabs used from July 22, 2020–June 18, 2021.

Similar proportions of testing events resulted in absence from work or school (43% vs. 49% for swabs with and without viral co-detection, respectively) and medical care seeking (16% vs. 24%) among those reporting any symptoms. Among those eligible and with known influenza vaccination status, 83% of specimens with and 76% of specimens without viral co-detection were from individuals that had received that current season's influenza vaccine at the time of sample collection. Due to the change in swab type during the study, 75% of specimens with *S. pneumoniae* and any virus detected were mid-turbinate swabs compared to 66% of specimens with only *S. pneumoniae* detected.

**Figure 1:**
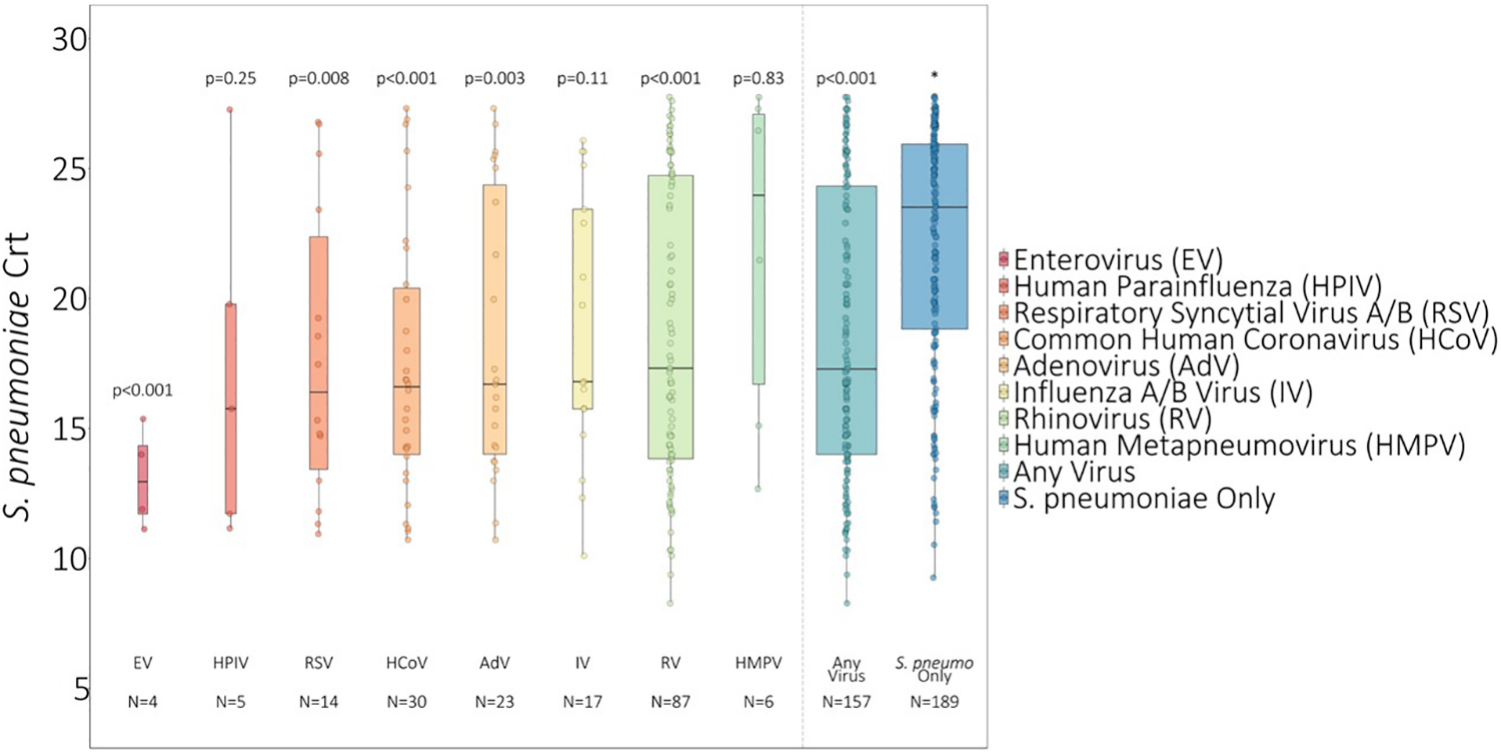
*S. pneumoniae* Crt values of swabs with and without respiratory viruses detected^1^. 1 Influenza A/B virus incudes *n* = 8 influenza A virus and *n* = 9 influenza B virus. RSV A/B includes *n* = 9 RSV A and *n* = 5 RSV B. * *P*-values comparing *S. pneumoniae* carriage Crt values with and without viral detection estimated using GEE of *S. pneumoniae* carriage Crt on virus detection adjusted for age and swab type and accounting for clustering of swabs within households. Width of boxplots corresponds to number of swabs.

**Figure 2:**
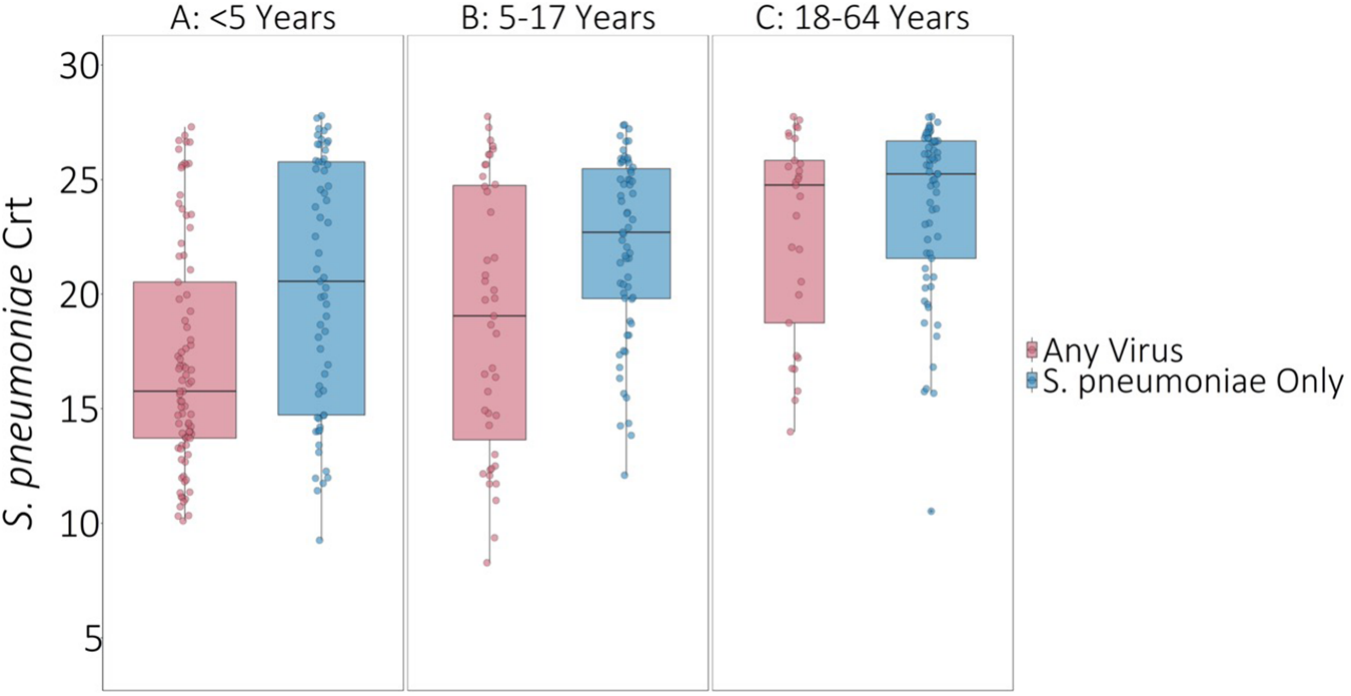
*S. pneumoniae* Crt values with and without respiratory viruses detected by age category. * *P*-values comparing *S. pneumoniae* carriage Crt values with and without viral detection estimated using GEE of *S. pneumoniae* carriage Crt on virus detection adjusted for age and swab type and accounting for clustering of swabs within households. Width of boxplots corresponds to number of swabs.

On average, *S. pneumoniae* Crt values were 2.67 units (95% CI: 1.55, 3.80, *P* < 0.001) lower (i.e., semiquantitative nasal carriage density was higher) for testing events with compared to without viral co-detection adjusted for age and swab type (Figure 1 and Supplementary Table S2). Of specific viruses evaluated, *S. pneumoniae* semiquantitative carriage density was higher with viral co-detection for enterovirus, RSV A/B, adenovirus, rhinovirus, and common human coronavirus compared to swabs without viral co-detection (*P* < 0.01 for each). No significant differences in *S. pneumoniae* semiquantitative carriage density were detected with or without influenza A/B virus, human parainfluenza and human metapneumovirus or between mid-turbinate and anterior nasal swabs. *S. pneumoniae* semiquantitative carriage density decreased significantly with age (*P* < 0.001). When analyses were stratified by age category, *S. pneumoniae* Crt values were significantly lower (corresponding to higher *S. pneumoniae* semiquantitative carriage density) with compared to without viral co-detection among children aged <5 years (2.52 units, 95% CI: 0.93, 4.11, *P* = 0.002) and 5–17 years (2.96 units, 0.90, 5.02, *P* = 0.005), but not among adults aged 18–64 years (1.08 units, −0.90, 3.05, *P* = 0.29) (Figure 2 and Supplementary Table S2).

Results were similar when restricting to symptomatic episodes. The only exception was human parainfluenza virus, where *S. pneumoniae* semiquantitative carriage density was significantly higher with human parainfluenza virus detection for specimens collected from symptomatic individuals (*P* < 0.001) but not for all specimens (*P* = 0.25), although among symptomatic individuals, human parainfluenza virus was only detected in one specimen. No difference in *S. pneumoniae* semiquantitative carriage density was detected by days since symptom onset (Supplementary Figure S5 and Supplementary Table S2). Results were also similar between the period prior to and post-implementation of COVID-19 restrictions and when excluding repeat swabs conducted by participants within 30 days (Supplementary Figures S6–S7 and Supplementary Table S2). No difference in *S. pneumoniae* semiquantitative carriage density was detected comparing individuals who had vs. had not received the current season influenza vaccine at the time of testing when comparing specimens with and without influenza A/B virus (Supplementary Table S2).

## Discussion

In a study of remote self-collection of nasal swab specimens in families with young children in the household setting, our analysis found that multiple common respiratory viruses were associated with greater concurrent *S. pneumoniae* semiquantitative nasal carriage density among children but not adults. Results were similar when restricting to specimens from symptomatic individuals. We also found that symptom profiles differed between individuals with specimens with and without viral co-detection, although we are unable to ascribe the etiology of the symptoms for individuals with swabs without any viruses detected. Overall, difficulty breathing, cough, and runny rose were reported more commonly among specimens with viral co-detection whereas ear pain and headache were reported less commonly among specimens with viral co-detection compared to specimens without. Although we tested for SARS-CoV-2, none of the SARS-CoV-2-positive specimens had *S. pneumoniae* co-detected.

Our findings of greater *S. pneumoniae* semiquantitative nasal carriage density with any viral co-detection among children are consistent with another US study in healthy children (30) and our findings for specific viruses are broadly consistent with other settings, although most studies evaluated only a few respiratory viruses. In this study, lower *S. pneumoniae* Crt values (corresponding to higher semiquantitative nasal carriage density) were associated with enterovirus, RSV A/B, adenovirus, rhinovirus, and common human coronavirus; we did not detect a difference for influenza A/B virus, human parainfluenza, or human metapneumovirus. Our findings for rhinovirus, adenovirus, and common human coronavirus are consistent with other studies which have also associated these viruses with increased pneumococcal carriage density (21, 27–29, 31, 32). Findings have been variable for RSV, which was associated with increased pneumococcal carriage density in this study, among hospitalized children in Vietnam (28), and among children with ARI in Kenya (29), but not among healthy children attending daycare in the UK (32). Influenza virus infection is a long-established risk factor for IPD (8, 9, 14, 22) but we did not find influenza A/B virus detection to be significantly associated with lower *S. pneumoniae* Crt values in this study. This finding for influenza virus is not consistent with the Vietnam study among hospitalized children (28) and the South Africa study among individuals of all ages hospitalized with ARI (21), but is consistent with the UK study of healthy children attending daycare, which found no association with influenza virus (32). Variability across studies, however, may be attributable to differences in study populations (e.g., healthy individuals vs. those with ARI) and sample collection methods (e.g., nasal vs. nasopharyngeal swabs). Similar to the UK study among children attending day care, we did not detect a difference in pneumococcal carriage density for human parainfluenza virus. None of these prior studies evaluated the association with enterovirus, which we found to be associated with higher semiquantative pneumococcal carriage density, or human metapneumovirus, which we did not find to be associated with semiquantative pneumococcal carriage density.

Our study found that viral co-detection was associated with increased *S. pneumoniae* semiquantitative nasal carriage density among children aged <5 years and 5–17 years, but not among adults aged 18–64 years and that increasing age was associated with lower *S. pneumoniae* semiquantitative nasal carriage density overall. One other study investigating *S. pneumoniae* carriage and viral co-detections among individuals of all ages hospitalized with ARI in South Africa found that adults had less pneumococcal nasopharyngeal colonization compared to children overall, but this study did not estimate the association between viral detection and carriage by age group. In addition, carriage and IPD serotype distributions, IPD disease burden, and pneumococcal vaccine uptake differ between the US and South Africa and thus these populations may not be directly comparable (21). The age effect observed in this study may be related to lower detection of *S. pneumoniae* among adults compared to children as *S. pneumoniae* carriage density is generally lower among adults and we used nasal rather than more sensitive nasopharyngeal swabs and did not collect oropharyngeal swabs among adults, as recommended by the WHO (41, 42). Alternatively, this may reflect differences in the processes leading to increased pneumococcal carriage between children and adults, a potential impact of a different serotype distribution between children and adults, differences in prior *S. pneumoniae* infection and vaccination histories, or differences in the distribution of respiratory viruses co-detected. Importantly, although eligible, our analysis did not include any adults aged ≥65 years and this pattern may not be consistent among older adults at higher risk for IPD (43).

This study was conducted over two years from November 2019–June 2021. Similar to other reports, we observed a COVID-19 pandemic nonpharmaceutical interventions-associated decline in respiratory viruses in 2020 and an out-of-season resurgence of respiratory viruses during the spring and summer months of 2021 while pneumococcal carriage declined to a lesser degree over the study period (44–49). This pattern was observed in both Israel and France, where surveillance data showed declines in the incidence of both respiratory viruses, such as RSV and influenza, and IPD following COVID-19 pandemic nonpharmaceutical interventions but no significant change in pneumococcal carriage rates, suggesting that the observed decline in IPD may be attributable to the decline in respiratory viruses rather than a change in pneumococcal carriage rates (44, 45). Our finding that RSV and common human coronavirus were associated with greater concurrent *S. pneumoniae* semiquantitative nasal carriage density is consistent with this hypothesis that infection with common respiratory viruses may increase risk for pneumococcal disease. However, enterovirus, adenovirus, and rhinovirus, which we found to be associated with greater concurrent *S. pneumoniae* semiquantitative nasal carriage density in this study, all persisted during the COVID-19 pandemic. Nonpharmaceutical interventions may have had a lesser impact on enterovirus, adenovirus, and rhinovirus due to these being non-enveloped viruses that are environmentally stable, have prolonged shedding, and are often asymptomatic or only mildly symptomatic (50). Therefore, the association between respiratory virus infection and pneumococcal disease risk may be pathogen-specific.

There were several limitations to our study. First, we cannot rule out that differences in pneumococcal carriage density found in our study are due to other underlying differences between individuals, such as genetic, immune, and environmental factors, including pneumococcal vaccination. In a study of children with ARI in Kenya where samples were taken before, during, and after viral infection, RSV and rhinovirus were associated with increased pneumococcal nasopharyngeal carriage density, but this variation was small relative to the variation in carriage density observed across individuals, leading the authors to conclude that viral co-infection may play only a small role in pneumococcal carriage density (29). Our study did not routinely collect more than one specimen per illness episode and thus we are unable to similarly estimate the variation in carriage density between individuals, evaluate changes in pneumococcal carriage density over time, or assess risk for subsequent pneumococcal disease. Second, symptom data were not collected from individuals at baseline or from household contacts of SARS-CoV-2 positive cases and thus we did not collect specimens from any individuals who confirmed not having any ARI symptoms at the time of sample collection. We therefore could not compare differences in the impact of viral co-detection on pneumococcal carriage density between symptomatic, pre-symptomatic, and asymptomatic individuals or between those with viral infection vs. those with viral detection. Our self-reported symptom data may also have underestimated symptoms that are not directly observable among young children. For example, ear pain and headache were infrequently reported among symptomatic children aged <5 years relative to older children. Third, we tested self-collected mid-turbinate and anterior nasal swabs rather than nasopharyngeal swabs, which are the current gold standard for detecting pneumococcal carriage in children, and we did not collect nasopharyngeal in addition to oropharyngeal swabs among adults, the current gold standard (41). Therefore, we likely underestimated detection of *S. pneumoniae*, such as in low *S. pneumoniae* density specimens (51). Indeed, our overall positivity rate for *S. pneumoniae* was very low (8%). In addition, rhinorrhea induced by viruses may have contributed to greater *S. pneumoniae* density in these relatively superficial samples. Specimen quality may have also been variable due to self-collection, however we have previously shown this method of self-collection to result in a high rate of adequate specimen quality (37). We also relied on *S. pneumoniae* Crt values as a proxy of semiquantitative carriage density rather than using the more precise gene copies/mL and our assay for detecting *S. pneumoniae* used proprietary primer sets and therefore data regarding the accuracy of the assay, including in comparison to real world nasopharyngeal samples with culture-based detection, are limited. Fourth, we switched from mid-turbinate to anterior nasal swabs for the second year of the study, although we were able to adjust for swab type in our analyses and did not find a difference in *S. pneumoniae* Crt values by swab type. Fifth, we tested for ten (and analyzed eight) common respiratory viruses and therefore cannot rule out that some swabs with *S. pneumoniae* only detected did not also contain a respiratory virus for which we did not test and cannot determine the etiology of symptoms for individuals with swabs without respiratory viruses detected. Some rhinovirus detections may also reflect enteroviruses due to cross-reactivity in our assay. Sixth, due to relatively small sample sizes we conducted stratified analyses for all viruses combined by age group rather than testing for interactions between age and viral co-detection in the main analysis. Seventh, our study population had relatively high rates of influenza vaccination and a very low rate of smoking and may not be generalizable to other populations. Eighth, our study included the period following implementation of nonpharmaceutical interventions to limit transmission of COVID-19, when expected seasonal peaks in respiratory viruses did not occur (50) and overall detection of viruses in the study was low, particularly during this period. Despite this, we did not find a meaningful difference in results when analyses were stratified by pre- and post-implementation of COVID-19 restrictions. Finally, *S. pneumoniae* positive specimens were not serotyped and we did not collect data on pneumococcal vaccination. Therefore, we could not assess the potential role of pneumococcal vaccination or specific serotypes on the association between viral co-detection and pneumococcal carriage density, an important area for continued study as some *S. pneumoniae* serotypes have higher invasiveness potential (52–56) and virus-*S. pneumoniae* associations may be serotype-specific (4, 18, 28, 57).

Despite these limitations, this longitudinal, community surveillance study included both children and adults aged <65 years of age, allowing us to evaluate the association between viral co-detection and pneumococcal carriage by age, which to our knowledge has not been previously studied for most respiratory viruses included in this study. In addition, our study was conducted in a household setting whereas the majority of prior studies evaluating the relationship between *S. pneumoniae* carriage and viral co-detection have been among hospitalized individuals. We also included data from the pre-COVID-19 period, during the COVID-19 pandemic, and during the out-of-season resurgence of respiratory viruses following the lifting of pandemic restrictions. Our data included eight common respiratory pathogens allowing for virus-specific estimates of the association between virus co-detection and pneumococcal carriage. Finally, we collected detailed data on symptom profiles, allowing comparisons of swabs with and without viral co-detection that is not possible for many studies relying on routinely collected surveillance and laboratory data.

In conclusion, several common respiratory viruses were associated with greater concurrent *S. pneumoniae* semiquantitative nasal carriage density among children but not adults before and during the COVID-19 pandemic in a community-based household setting. These findings suggest that some common respiratory viruses may increase risk of subsequent pneumococcal disease in children. The potential impact of vaccines against respiratory viruses, including influenza vaccines and future RSV vaccines, for preventing pneumococcal disease should be explored.

## Data Availability

The raw data supporting the conclusions of this article will be made available by the authors, without undue reservation.
