# Supplementary material for: Streptococcus pneumoniae nasal carriage patterns with and without common respiratory virus detections in households in Seattle, WA, USA before and during the COVID-19 pandemic
Source: Front Pediatr. 2023 Jul 7;11:1198278. doi: 10.3389/fped.2023.1198278 (PMC10361771; doi:10.3389/fped.2023.1198278)
Supplement: Supplementary file 1 [file Datasheet.pdf]

Streptococcus pneumoniae Nasal Carriage Patterns with and without Common Respiratory Virus Detections in Households in Seattle, WA, USA Before and During the COVID-19 Pandemic

Supplementary Materials

Table of Contents

Supplementary Figure 1. Detection of *S. pneumoniae* or respiratory viruses over time for all swabs collected (14-day moving averages)………………..……….……………………1

Supplementary Figure 2. Detection of *S. pneumoniae* or respiratory viruses over time for all swabs collected by age group (14-day moving averages) ……………….…………..…2

Supplementary Figure 3. Distribution of viruses co-detected with *S. pneumoniae* by age group………………………………………………………………………………………3

Supplementary Figure 4. Symptoms reported at swab collection among *S. pneumoniae*-positive swabs with and without respiratory viruses co-detected among individuals reporting any symptoms……………………………………………………..………….4

Supplementary Table 1. Characteristics of testing events and symptoms by age group..5

Supplementary Figure 5. *S. pneumoniae* Crt values with and without respiratory viruses detected among symptomatic testing events……………………...………………………6

Supplementary Figure 6. *S. pneumoniae* Crt values with and without respiratory viruses detected by pre- and post-implementation of COVID-19 restrictions (beginning in March 2020)………………………….…….………………….…………………….……………..…7

Supplementary Figure 7. *S. pneumoniae* Crt values of swabs with and without respiratory viruses detected, excluding repeat swabs conducted by participants within 30 days…………………………….….………………….…………………….……………....8

Supplementary Table 2. Linear generalized estimating equations (GEE) of *S. pneumoniae* carriage Crt on virus detection accounting for clustering of swabs within households …...…………………………………………………………..……………9–10

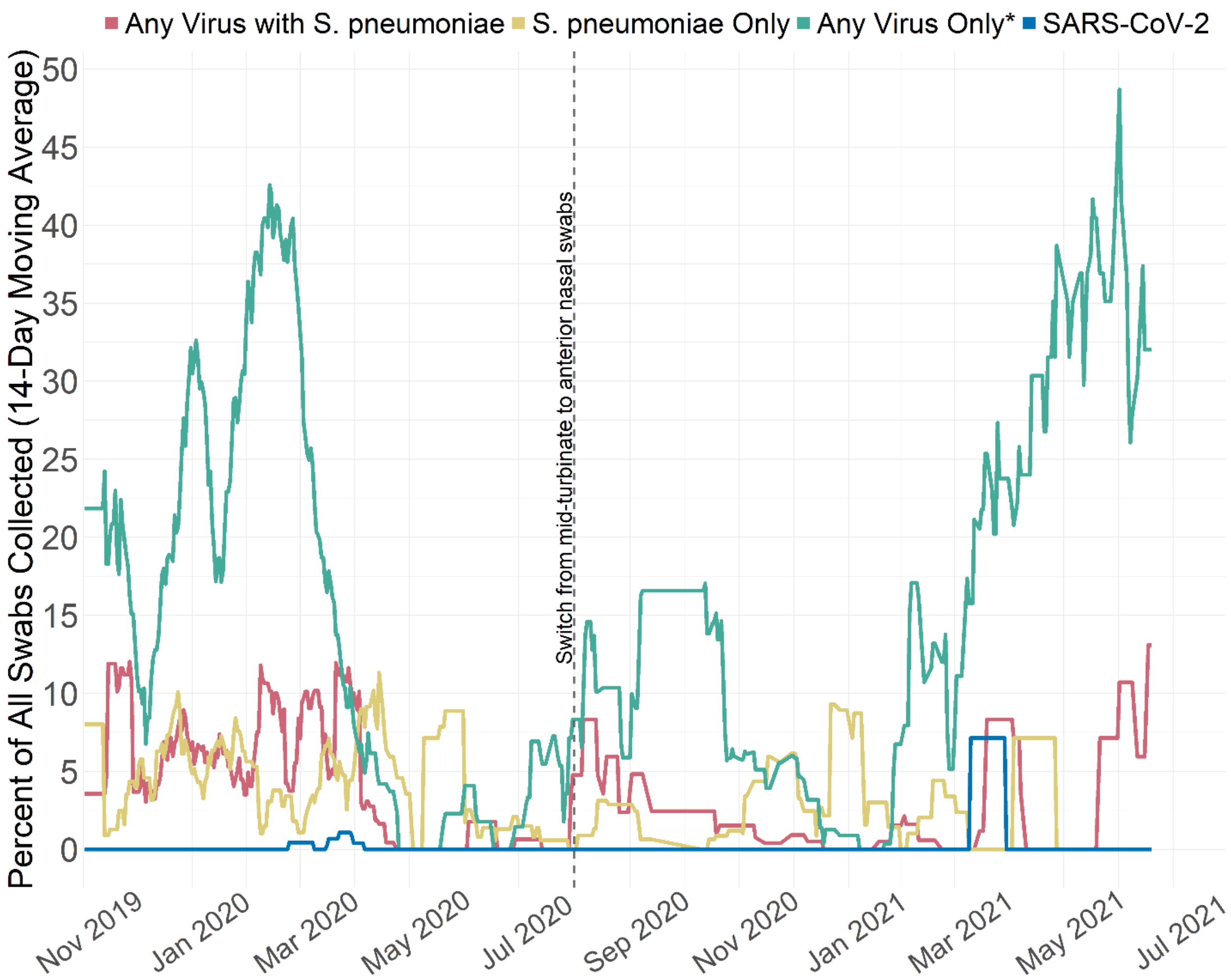

Supplementary Figure 1. Detection of *S. pneumoniae* or respiratory viruses over time for all swabs collected (14-day moving averages), excluding swabs collected from household contacts of SARS-CoV-2 positive cases (which were only collected in the second year of the study)

* Any virus only excludes those with only SARS-CoV-2 detected. SARS-CoV-2 was detected in 35 specimens, none of which had *S. pneumoniae* co-detected

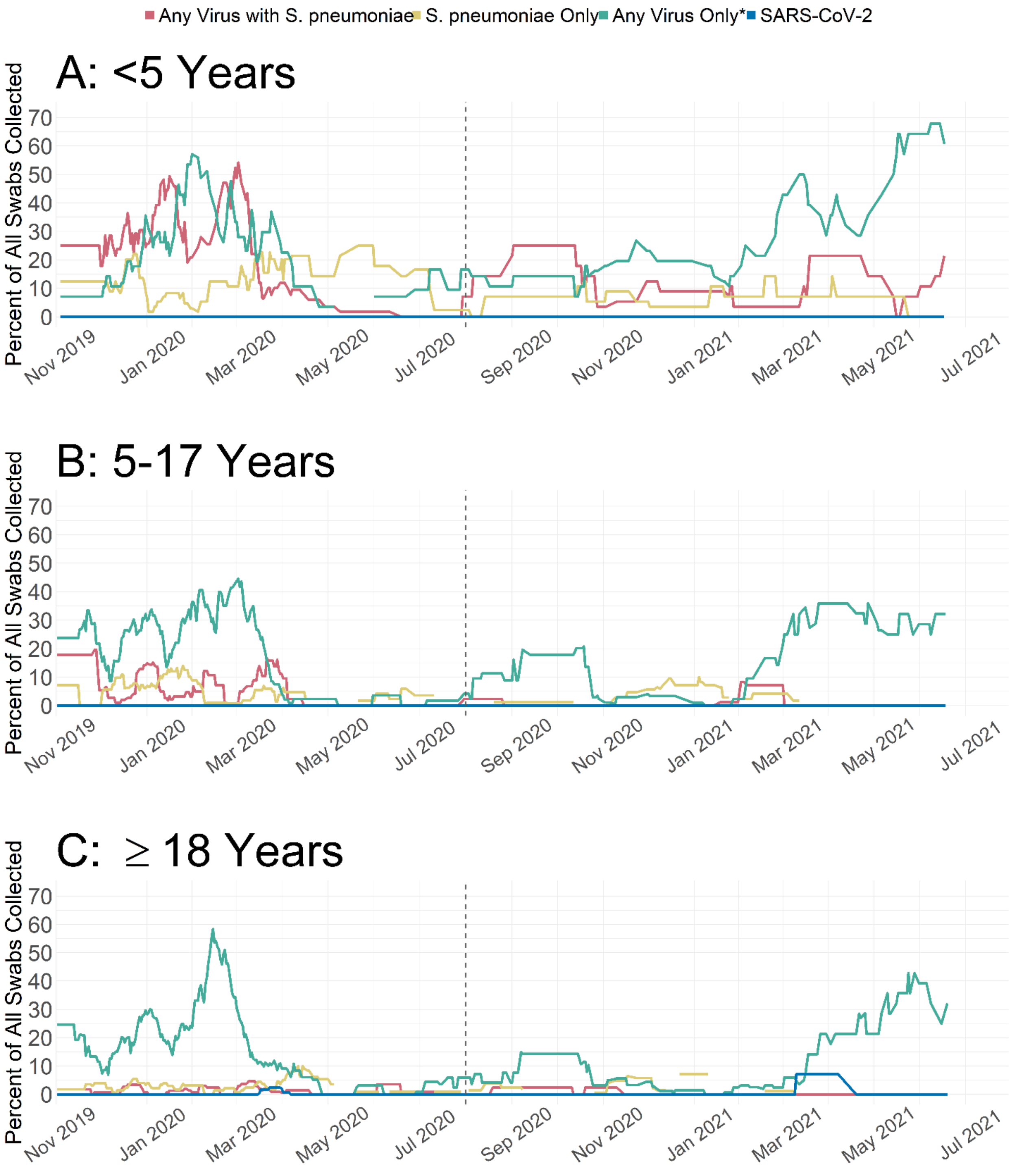

Supplementary Figure 2. Detection of S. pneumoniae or respiratory viruses over time for all swabs collected by age group (14-day moving averages), excluding swabs collected from household contacts of SARS-CoV-2 positive cases (which were only collected in the second year of the study)

* Any virus only excludes those with only SARS-CoV-2 detected
Vertical dashed line indicates switch from mid-turbinate to anterior nasal swabs

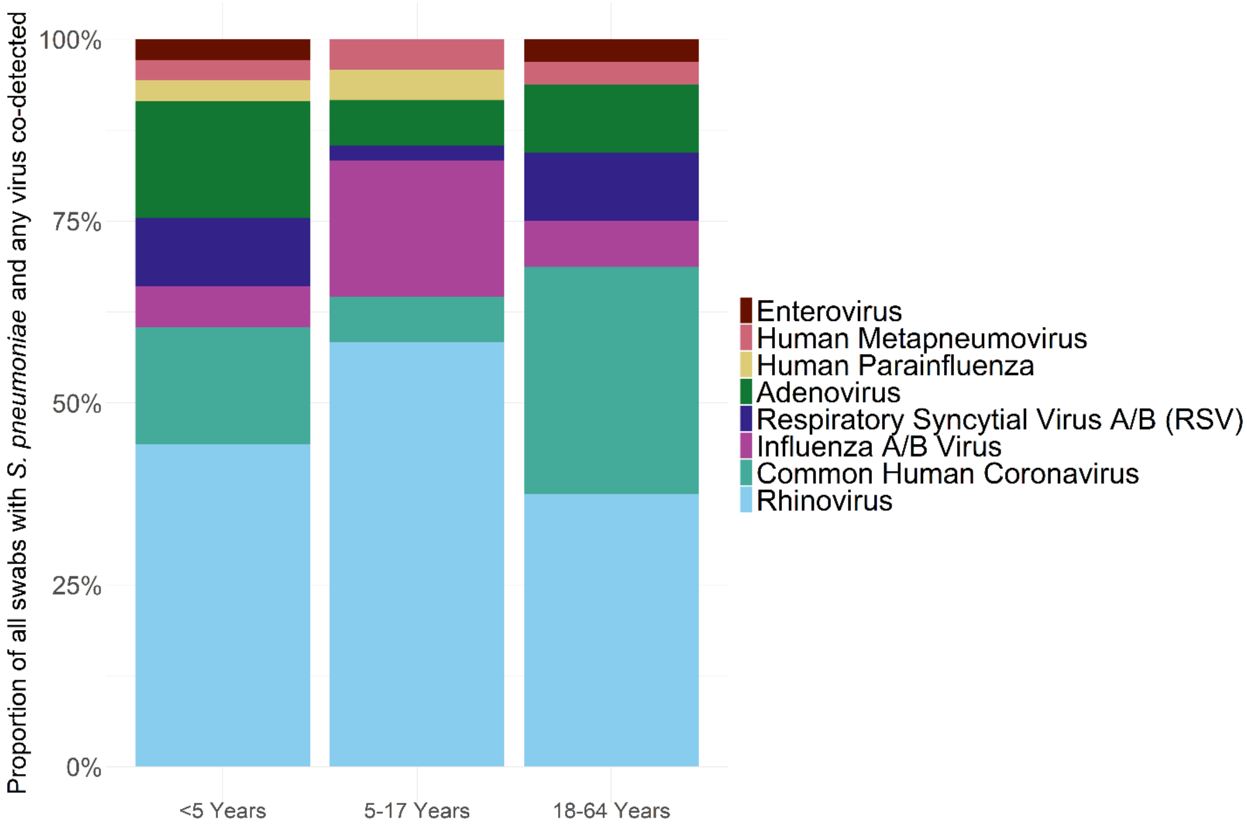

Supplementary Figure 3. Distribution of viruses co-detected with *S. pneumoniae* by age group

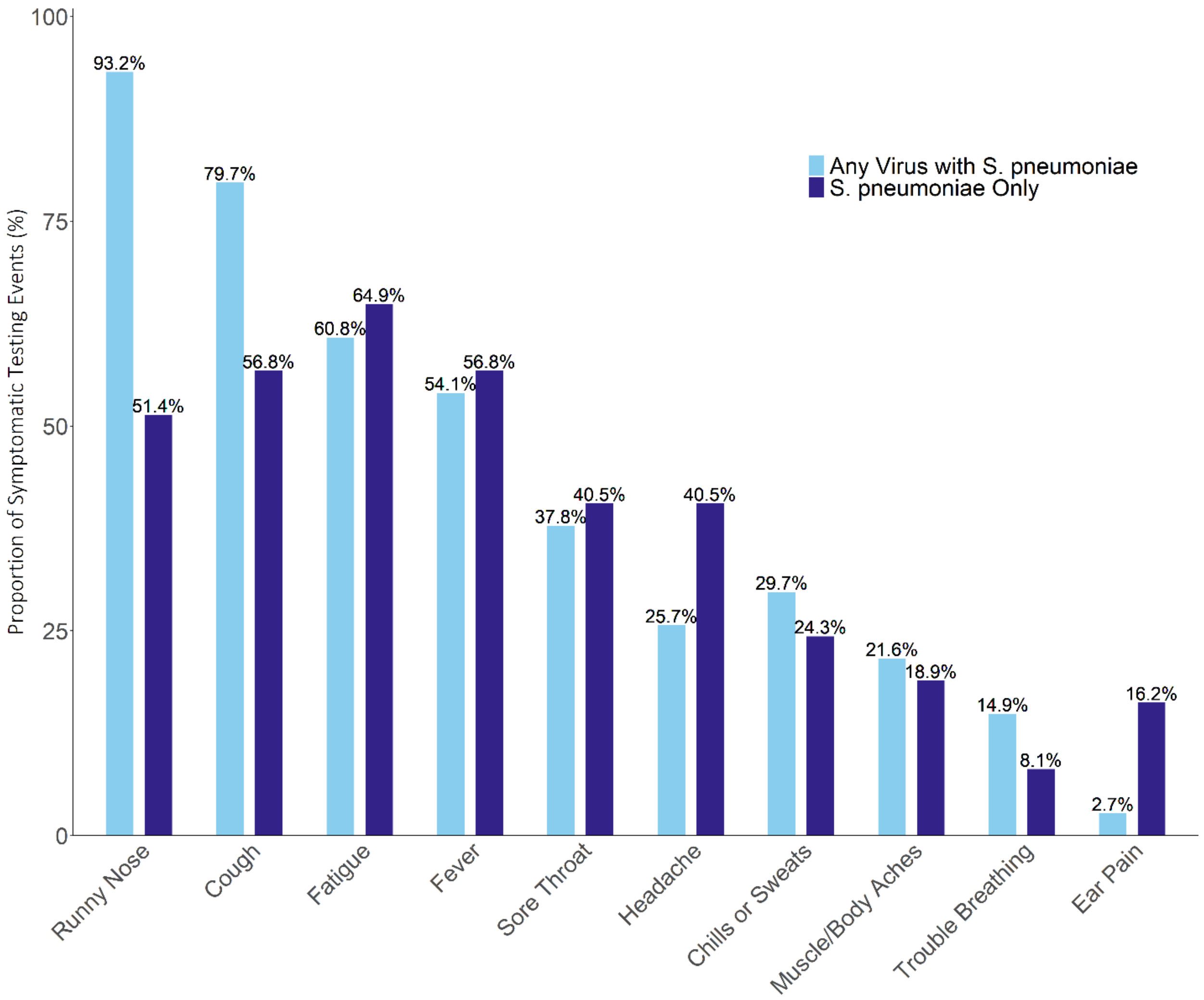

Supplementary Figure 4. Symptoms reported at swab collection among *S. pneumoniae*-positive swabs with and without respiratory viruses co-detected among individuals reporting any symptoms (N=111, 32% of all *S. pneumoniae*-positive swabs included in analysis)

Supplementary Table 1. Characteristics of symptoms at self-swab collection by age group

	Children <5 years (N=146)	Children 5–17 years (N=106)	Adults 18–64 years (N=94)
**Symptomatic**, N (%)			
Yes	51 (34.9%)	35 (33.0%)	25 (26.6%)
Days since symptom onset, median (IQR[1])	3 (2, 4)	3 (2, 4)	2 (2, 3)
Influenza-like illness (ILI)[2,3]	22 (43.1%)	20 (57.1%)	7 (28.0%)
Difficulty breathing[2]	6 (11.8%)	2 (5.7%)	6 (24.0%)
Chills or sweats[2]	13 (25.5%)	12 (34.3%)	6 (24.0%)
Cough[2]	38 (74.5%)	27 (77.1%)	15 (60.0%)
Ear pain[2]	1 (2.0%)	4 (11.4%)	3 (12.0%)
Feeling more tired than usual[2]	24 (47.1%)	26 (74.3%)	19 (76.0%)
Fever/feeling feverish[2]	30 (58.8%)	22 (62.9%)	9 (36.0%)
Headache[2]	3 (5.9%)	14 (40.0%)	17 (68.0%)
Muscle and/or body aches[2]	5 (9.8%)	8 (22.9%)	10 (40.0%)
Runny nose[2]	46 (90.2%)	25 (71.4%)	17 (68.0%)
Sore throat[2]	8 (15.7%)	20 (57.1%)	15 (60.0%)
Unknown[4]	95 (65.1%)	71 (67.0%)	69 (73.4%)

1 Interquartile range
2 Proportions for specific symptoms, absence from work or school, and sought medical care are among those reporting any symptoms
3 ILI includes fever with cough and/or sore throat
4 Swabs with unknown symptom status include baseline enrollment swabs and swabs from household contacts after SARS-CoV-2 was detected in a household member (in the second year of the study only

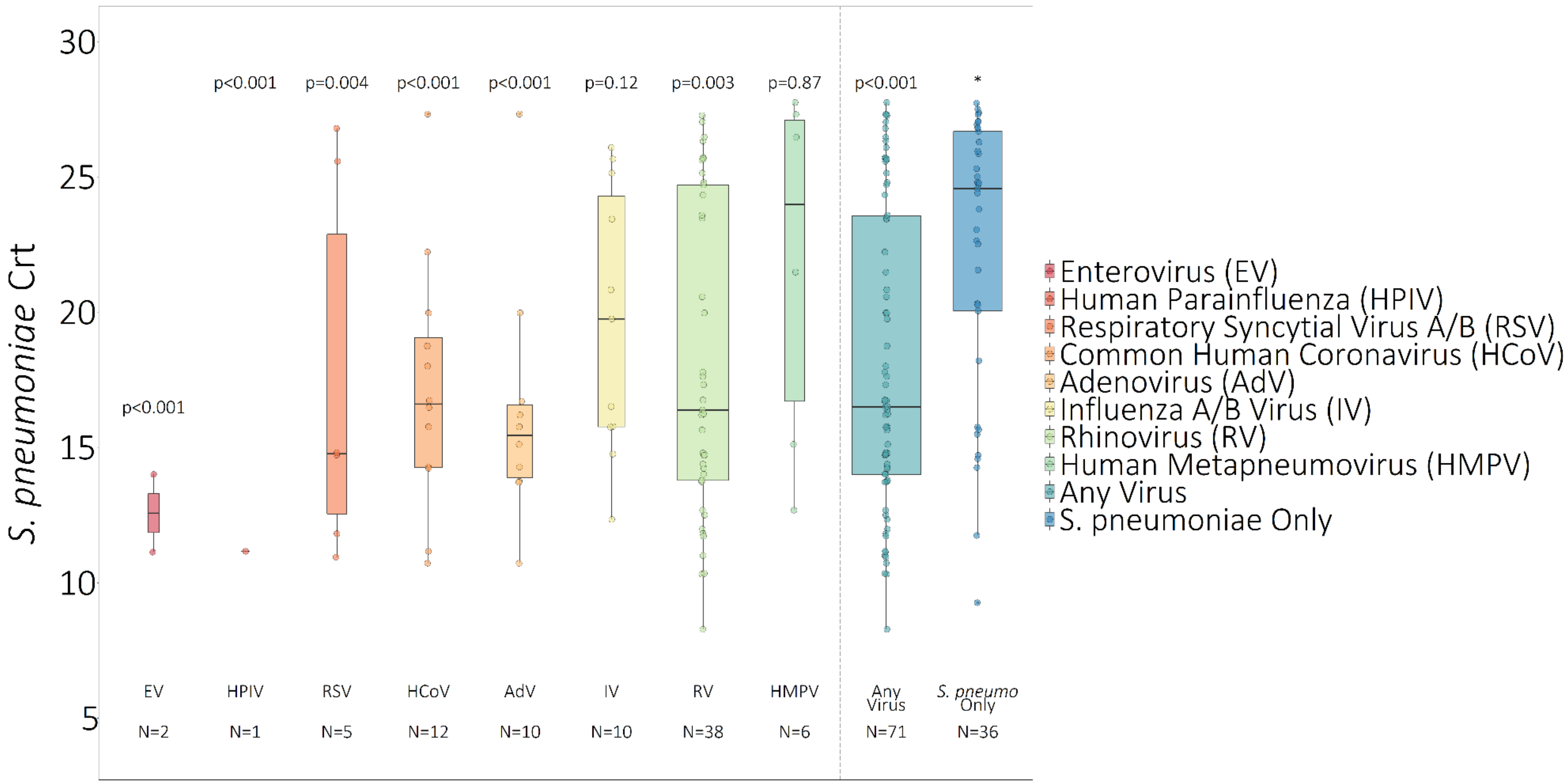

Supplementary Figure 5. *S. pneumoniae* Crt values of swabs with and without respiratory viruses detected among symptomatic testing events symptoms (N=107, 32% of all *S. pneumoniae*-positive swabs from N=82, 34% of all participants included in analysis)[1]

1 Analysis of symptomatic testing events excluded one swab with an implausible symptom onset date (>100 days prior to testing) and three swabs with symptom onset after swab collection

* P-values comparing *S. pneumoniae* carriage Crt values of swabs with and without viral detection for symptomatic testing events estimated using GEE of *S. pneumoniae* carriage Crt on virus detection adjusted for age, swab type, and days since symptom onset and accounting for clustering of swabs within households. Width of boxplots corresponds to number of swabs

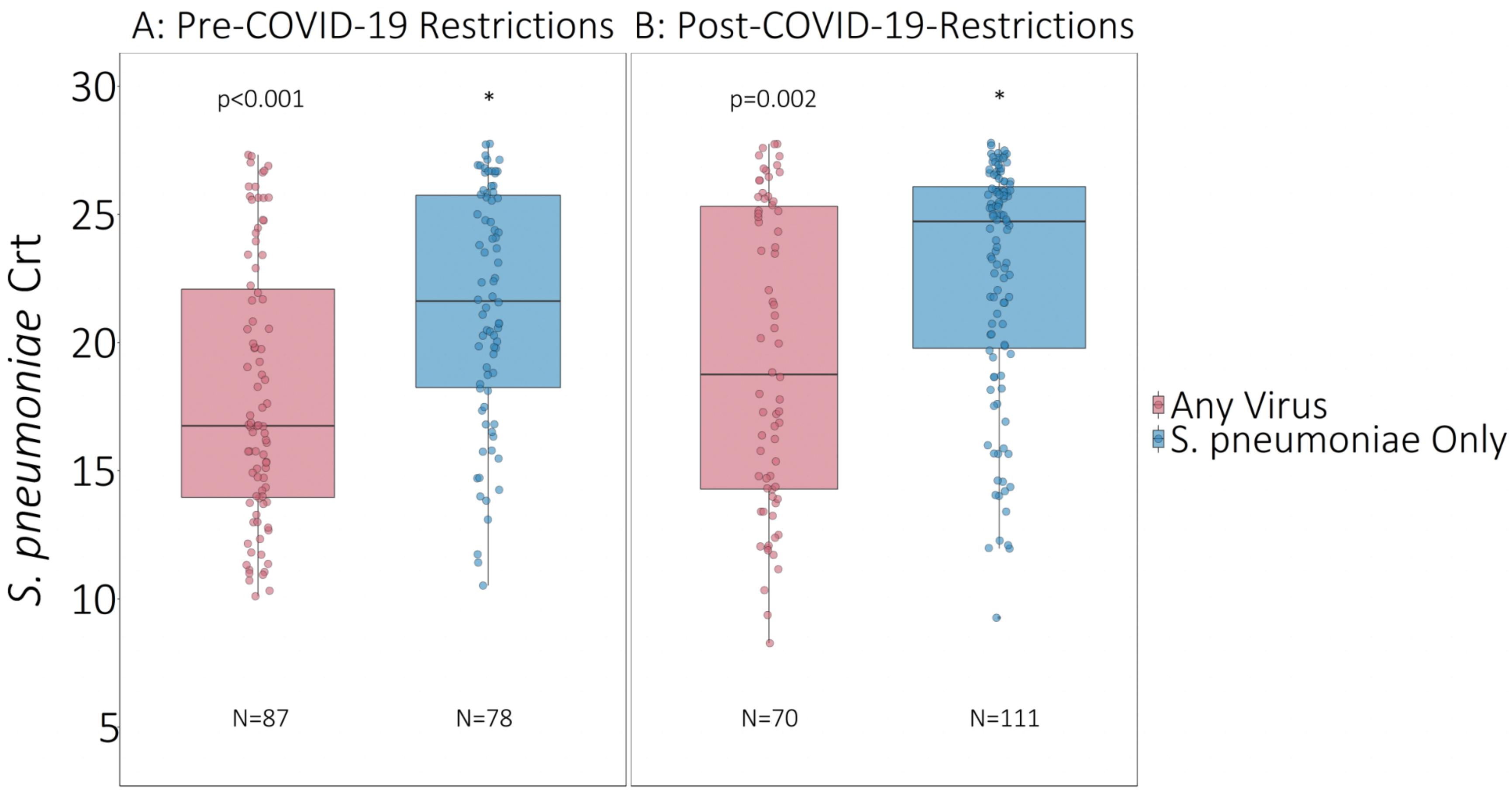

Supplementary Figure 6. *S. pneumoniae* Crt values of swabs with and without respiratory viruses detected by pre- (prior to March 2020) and post-implementation of COVID-19 restrictions (March 2020 onward)

* P-values comparing *S. pneumoniae* carriage Crt values of swabs with and without viral detection estimated using GEE of *S. pneumoniae* carriage Crt on virus detection adjusted for age and swab type (for post-COVID-19 restrictions period only as all were all were mid-turbinate nasal swabs prior to March 2020) and accounting for clustering of swabs within households. Width of boxplots corresponds to number of swabs

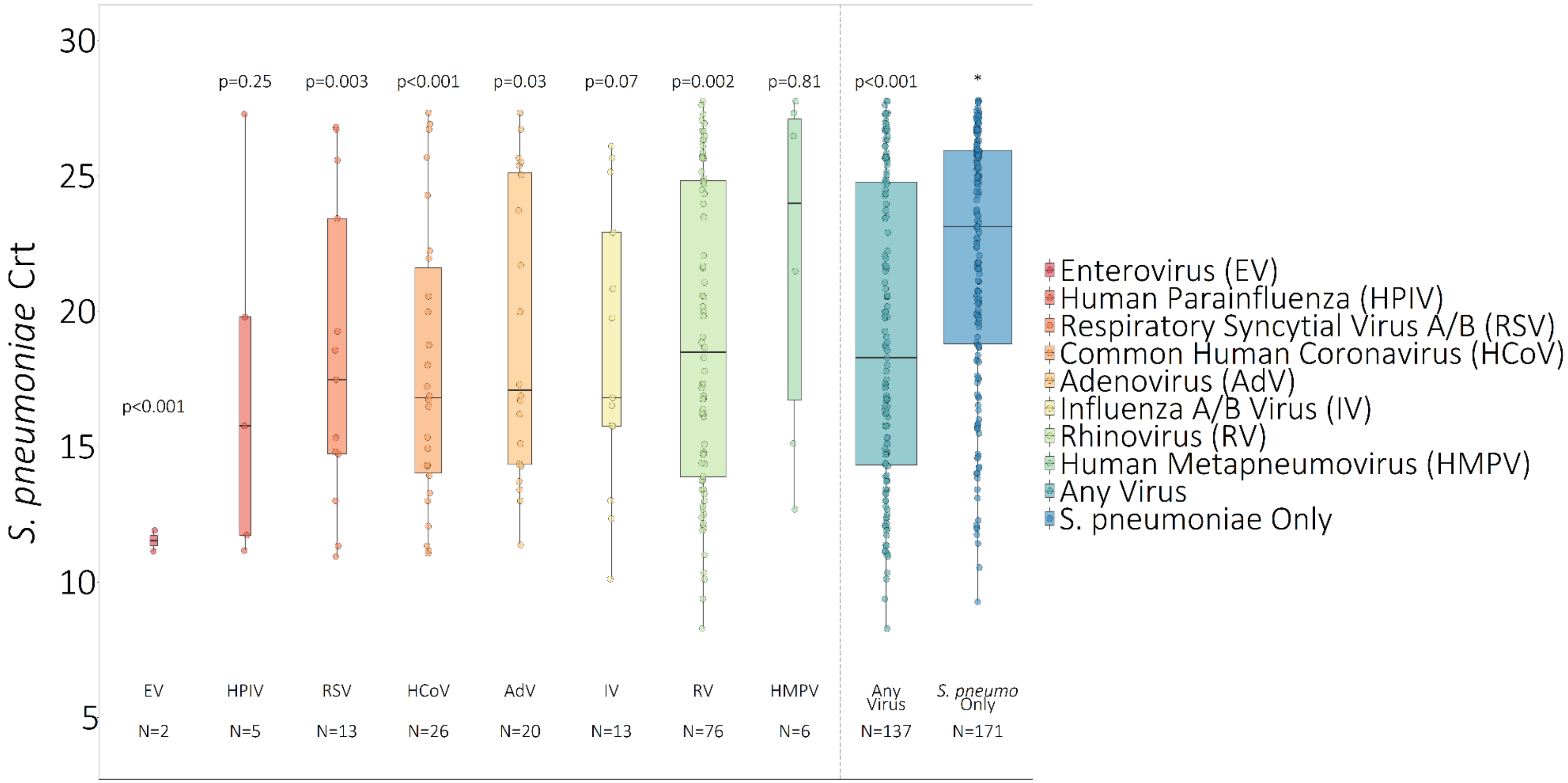

Supplementary Figure 7. *S. pneumoniae* Crt values of swabs with and without respiratory viruses detected, excluding repeat swabs conducted by participants within 30 days (N=308, 89% of all *S. pneumoniae*-positive swabs)

* P-values comparing *S. pneumoniae* carriage Crt values of swabs with and without viral detection estimated using GEE of *S. pneumoniae* carriage Crt on virus detection adjusted for age and swab type and accounting for clustering of swabs within households. Width of boxplots corresponds to number of swabs

Supplementary Table 2. Linear generalized estimating equations (GEE) of *S. pneumoniae* carriage Crt on virus detection accounting for clustering of swabs within households

	**Mean adjusted difference in *S. pneumoniae* Crt value (95% CI)**	**p-value**
**All swabs, adjusted for age and swab type**		
S. pneumoniae only (n=189) vs. any virus (n=157)	2.67 (1.55, 3.80)	**<0.001**
vs. Enterovirus (n=4)	8.87 (7.15, 10.59)	**<0.001**
vs. RSV A/B (n=14)	3.08 (0.80, 5.36)	**0.008**
vs. Adenovirus (n=23)	2.59 (0.91, 4.27)	**0.003**
vs. Rhinovirus (n=87)	2.78 (1.29, 4.27)	**<0.001**
vs. Common Human Coronavirus (n=30)	4.11 (2.69, 5.54)	**<0.001**
vs. Influenza A/B Virus (n=17)	2.05 (-0.47, 4.57)	0.11
vs. Human Parainfluenza (n=5)	3.70 (-2.60, 9.99)	0.25
vs. Human Metapneumovirus (n=6)	-0.51 (-5.12, 4.09)	0.83
Age (years)	0.10 (0.07, 0.14)	**<0.001**
Swab type (mid-turbinate vs. anterior nasal)	-1.09 (-2.28, 0.11)	0.07
**Symptomatic testing events, adjusted for age, swab type, and days since symptom onset[1]**		
S. pneumoniae only (n=36) vs. any virus (n=71)	3.46 (1.80, 5.12)	**<0.001**
vs. Enterovirus (n=2)	7.90 (5.31, 10.49)	**<0.001**
vs. RSV A/B (n=5)	4.97 (1.55, 8.39)	**0.004**
vs. Adenovirus (n=10)	4.53 (2.49, 6.57)	**<0.001**
vs. Rhinovirus (n=38)	3.23 (1.11, 5.35)	**0.003**
vs. Common Human Coronavirus (n=12)	5.10 (3.24, 6.96)	**<0.001**
vs. Influenza A/B Virus (n=10)	2.80 (-0.70, 6.30)	0.12
vs. Human Parainfluenza (n=1)	11.14 (7.83, 14.46)	**<0.001**
vs. Human Metapneumovirus (n=6)	0.40 (-4.41, 5.21)	0.87
Age (years)	0.15 (0.08, 0.23)	**<0.001**
Swab type (mid-turbinate vs. anterior nasal)	-1.33 (-4.06, 1.40)	0.34
Days since symptom onset	0.30 (-0.19, 0.78)	0.23
**All swabs, excluding repeat swabs conducted by participants within 30 days, adjusted for age and swab type**		
S. pneumoniae only (n=171) vs. any virus (n=137)	2.32 (1.19, 3.47)	**<0.001**
vs. Enterovirus (n=2)	9.78 (8.44, 11.12)	**<0.001**
vs. RSV A/B (n=13)	2.55 (0.29, 4.82)	**0.003**
vs. Adenovirus (n=20)	1.98 (0.23, 3.73)	**0.03**
vs. Rhinovirus (n=76)	2.31 (0.86, 3.76)	**0.002**
vs. Common Human Coronavirus (n=26)	3.73 (2.10, 5.41)	**<0.001**
vs. Influenza A/B Virus (n=13)	2.41 (-0.35, 5.18)	0.07
vs. Human Parainfluenza (n=5)	3.72 (-2.66, 10.11)	0.25
vs. Human Metapneumovirus (n=6)	-0.56 (-5.12, 4.07)	0.81
Age (years)	0.10 (0.07, 0.13)	**<0.001**
Swab type (mid-turbinate vs. anterior nasal)	-1.33 (-2.50, -0.16)	**0.03**
**Children aged <5 years, adjusted for age and swab type**		
S. pneumoniae only (n=61) vs. any virus (n=85)	2.52 (0.93, 4.11)	**0.002**
Age (years)	0.96 (0.19, 1.72)	**0.014**
Swab type (mid-turbinate vs. anterior nasal)	-2.36 (-4.27, -0.45)	**0.016**
**Children aged 5-17 years, adjusted for age and swab type**		

S. pneumoniae only (n=63) vs. any virus (n=43)	2.96 (0.90, 5.02)	**0.005**
Age (years)	0.20 (-0.19, 0.58)	0.31
Swab type (mid-turbinate vs. anterior nasal)	0.19 (-2.00, 2.38)	0.86
**Adults aged 18-64 years, adjusted for age and swab type**		
S. pneumoniae only (n=65) vs. any virus (n=29)	1.08 (-0.90, 3.05)	0.29
Age (years)	0.02 (-0.17, 0.20)	0.87
Swab type (mid-turbinate vs. anterior nasal)	-0.39 (-2.34, 1.55)	0.69
**Swabs collected prior to March 1, 2020, adjusted for age[2]**		
S. pneumoniae only (n=78) vs. any virus (n=87)	2.67 (1.20, 4.14)	**<0.001**
Age (years)	0.07 (0.02, 0.17)	**<0.001**
**Swabs collected on or after March 1, 2020, adjusted for age and swab type**		
S. pneumoniae only (n=111) vs. any virus (n=70)	2.58 (0.94, 4.23)	**0.002**
Age (years)	0.09 (0.04, 0.13)	**<0.001**
Swab type (mid-turbinate vs. anterior nasal)	-0.72 (-2.31, 0.87)	0.37
**Swabs with influenza A/B virus detected and known current season influenza vaccination status, adjusted for age, swab type, and influenza vaccination**		
S. pneumoniae only (n=177) vs. Influenza A/B Virus (n=17)	2.31 (-0.17, 4.79)	0.06
Age (years)	0.07 (0.03, 0.11)	**<0.001**
Swab type (mid-turbinate vs. anterior nasal)	-1.74 (-3.02,- 0.45)	**0.008**
Influenza vaccination at time of testing (received vs. not received)	0.31 (-1.33, 1.94)	0.71

1 Analysis of symptomatic testing events excluded one swab with an implausible symptom onset date (>100 days prior to testing) and three swabs with symptom onset after swab collection
2 Analysis of swabs prior to March 2020 not adjusted for swab type because all were mid-turbinate nasal swabs
